# Epcoritamab, a Novel, Subcutaneous CD3xCD20 Bispecific T-Cell–Engaging Antibody, in Relapsed or Refractory Large B-Cell Lymphoma: Dose Expansion in a Phase I/II Trial

**DOI:** 10.1200/JCO.22.01725

**Published:** 2022-12-22

**Authors:** Catherine Thieblemont, Tycel Phillips, Herve Ghesquieres, Chan Y. Cheah, Michael Roost Clausen, David Cunningham, Young Rok Do, Tatyana Feldman, Robin Gasiorowski, Wojciech Jurczak, Tae Min Kim, David John Lewis, Marjolein van der Poel, Michelle Limei Poon, Mariana Cota Stirner, Nurgul Kilavuz, Christopher Chiu, Menghui Chen, Mariana Sacchi, Brian Elliott, Tahamtan Ahmadi, Martin Hutchings, Pieternella J. Lugtenburg

**Affiliations:** ^1^ Assistance Publique & Hôpitaux de Paris (APHP), Hôpital Saint-Louis, Hémato-oncologie, Université de Paris, Paris, France; ^2^ University of Michigan Comprehensive Cancer Center, Ann Arbor, MI; ^3^ Hospices Civils de Lyon, Centre Hospitalier Lyon Sud, Pierre-Bénite, France; ^4^ Sir Charles Gairdner Hospital, Perth, Australia; ^5^ Division of Internal Medicine, Medical School, University of Western Australia, Perth, Australia; ^6^ Vejle Hospital, Vejle, Denmark; ^7^ The Royal Marsden NHS Foundation Trust, Sutton, United Kingdom; ^8^ Keimyung University Dongsan Medical Center, Daegu, Republic of Korea; ^9^ Hackensack Meridian Health Hackensack University Medical Center, Hackensack, NJ; ^10^ Concord Hospital, University of Sydney, Sydney, Australia; ^11^ MSC National Research Institute of Oncology, Kraków, Poland; ^12^ Seoul National University Hospital, Seoul, Republic of Korea; ^13^ University Hospitals Plymouth NHS Trust, Derriford Hospital, Plymouth, United Kingdom; ^14^ On behalf of the Lunenburg Lymphoma Phase I/II Consortium-HOVON/LLPC, Maastricht, Department of Internal Medicine, Division of Hematology, GROW School for Oncology and Developmental Biology, Maastricht University Medical Center, Maastricht, the Netherlands; ^15^ National University Hospital, Singapore; ^16^ AbbVie, North Chicago, IL; ^17^ Genmab, Princeton, NJ; ^18^ Rigshospitalet, Copenhagen University Hospital, Copenhagen, Denmark; ^19^ On behalf of the Lunenburg Lymphoma Phase I/II Consortium-HOVON/LLPC, Erasmus MC Cancer Institute, University Medical Center, Department of Hematology, Rotterdam, the Netherlands

## Abstract

Epcoritamab is a subcutaneously administered CD3xCD20 T-cell–engaging, bispecific antibody that activates T cells, directing them to kill malignant CD20^+^ B cells. Single-agent epcoritamab previously demonstrated potent antitumor activity in dose escalation across B-cell non-Hodgkin lymphoma subtypes.

**PATIENTS AND METHODS:**

In the dose-expansion cohort of a phase I/II study (ClinicalTrials.gov identifier: NCT03625037), adults with relapsed or refractory CD20^+^ large B-cell lymphoma and at least two prior therapy lines (including anti-CD20 therapies) received subcutaneous epcoritamab in 28-day cycles (once weekly step-up doses in weeks 1-3 of cycle 1, then full doses once weekly through cycle 3, once every 2 weeks in cycles 4-9, and once every 4 weeks in cycle 10 and thereafter) until disease progression or unacceptable toxicity. The primary end point was overall response rate by the independent review committee.

**RESULTS:**

As of January 31, 2022, 157 patients were treated (median age, 64 years [range, 20‐83]; median of three [range, 2-11] prior therapy lines; primary refractory disease: 61.1%; prior chimeric antigen receptor (CAR) T-cell exposure: 38.9%). At a median follow-up of 10.7 months, the overall response rate was 63.1% (95% CI, 55.0 to 70.6) and the complete response rate was 38.9% (95% CI, 31.2 to 46.9). The median duration of response was 12.0 months (among complete responders: not reached). Overall and complete response rates were similar across key prespecified subgroups. The most common treatment-emergent adverse events were cytokine release syndrome (49.7%; grade 1 or 2: 47.1%; grade 3: 2.5%), pyrexia (23.6%), and fatigue (22.9%). Immune effector cell–associated neurotoxicity syndrome occurred in 6.4% of patients with one fatal event.

**CONCLUSION:**

Subcutaneous epcoritamab resulted in deep and durable responses and manageable safety in highly refractory patients with large B-cell lymphoma, including those with prior CAR T-cell exposure.

## INTRODUCTION

Large B-cell lymphoma (LBCL) is a heterogeneous group of hematologic malignancies.^1,2^ Although new therapies have become available, management of relapsed or refractory LBCL remains a challenge.^3^ Outcomes are poor, particularly among patients with early relapse or primary refractory disease. Response rates range from 20% to 39%, and the median overall survival (OS) was 6.3 months in 636 patients with LBCL who relapsed or were refractory to first-line chemoimmunotherapy.^3^ Several therapies are approved in the United States, including polatuzumab vedotin in combination with bendamustine and rituximab, tafasitamab in combination with lenalidomide, loncastuximab tesirine, selinexor, and chimeric antigen receptor (CAR) T-cell therapies. CAR T-cell therapy represents a major advancement; however, consistency of bioengineering, manufacturing timelines, and access are limited globally.^4,5^ Thus, an unmet medical need still remains for effective, well-tolerated, and convenient therapies.

**CONTEXT**

**Key Objective**
Outcomes are poor for patients with relapsed or refractory large B-cell lymphoma (LBCL). This study evaluated the efficacy and safety of subcutaneous epcoritamab, a bispecific antibody targeting CD3 and CD20, in a dose-expansion cohort of patients with relapsed or refractory LBCL.
**Knowledge Generated**
Single-agent epcoritamab demonstrated high overall response rates, including deep and durable complete responses. Adverse events were manageable, with few discontinuations; cytokine release syndrome was mostly low grade with predictable timing.
**Relevance *(J.W. Friedberg)***
The observed high response rates with durability in patients with refractory LBCL treated with epcoritamab, including post–chimeric antigen receptor-T therapy, represent further evidence of the value of bispecific antibodies in this setting. Future studies need to evaluate feasibility of limited duration therapy, explore rational combinations, and incorporate this agent into earlier lines of treatment.*
*Relevance section written by *JCO* Editor-in-Chief Jonathan W. Friedberg, MD.

Epcoritamab (GEN3013) is a subcutaneously administered, bispecific antibody targeting CD3 and CD20 that redirects and activates T cells to kill CD20-expressing malignant cells.^6^ In preclinical evaluation, epcoritamab resulted in potent and selective T-cell–mediated cytotoxic activity against CD20^+^ malignant B cells.^6,7^ The dose-escalation portion met the primary end point, with the recommended phase II dose established and no dose-limiting toxicity in patients with relapsed or refractory CD20^+^ mature B-cell non-Hodgkin lymphoma.^8^ Here, we report results from the LBCL expansion cohort.

## METHODS

### Study Design and Patients

This was a phase I/II, single-arm, multicenter, open-label, dose-escalation/dose-expansion study in patients with relapsed, progressive, and/or refractory mature B-cell lymphoma (EPCORE NHL-1; GCT3013-01; ClinicalTrials.gov identifier: NCT03625037).

Eligible patients were at least age 18 years with an Eastern Cooperative Oncology Group performance status of 0 to 2 and documented CD20^+^ mature B-cell neoplasm (diffuse large B-cell lymphoma [DLBCL] or other aggressive non-Hodgkin lymphoma, including primary mediastinal LBCL, high-grade B-cell lymphoma, or follicular lymphoma grade 3B).^1^ Other inclusion criteria were relapsed or refractory disease, treatment with at least two prior lines of systemic therapy, including at least one anti-CD20–containing regimen, and prior failure or ineligibility for autologous stem-cell transplantation. Relapsed disease was defined as recurrence at least 6 months after completion of therapy, and refractory disease was defined as progression either during therapy or within 6 months of completion of therapy. Patients with prior CAR T-cell therapy were eligible (if ≥ 30 days since last treatment). There were no requirements for minimum life expectancy or absolute leukocyte count. Full inclusion and exclusion criteria are given in the Data Supplement (online only).

Patients received subcutaneous epcoritamab with cycle 1 step-up dosing consisting of a 0.16-mg priming dose once on day 1, followed by a 0.8-mg intermediate dose once on day 8, and subsequent full 48-mg doses once on day 15 and beyond until disease progression or unacceptable toxicity. Epcoritamab was administered as a 1-mL injection once weekly in cycles 1-3, once every 2 weeks during cycles 4-9 (days 1 and 15), and once every 4 weeks from cycle 10. No initial B-cell–depleting treatment was administered. During cycle 1, prophylaxis for cytokine release syndrome (CRS) included prednisolone 100 mg orally (or intravenous equivalent) administered 30-120 minutes before each epcoritamab dose (once daily on days 1-4 for the priming dose, once daily on days 8-11 for the intermediate dose, once daily on days 15-18 for the first full dose, and once daily on days 22-25 for the second full dose). In addition, diphenhydramine 50 mg orally or intravenously (or equivalent) and acetaminophen 650-1,000 mg orally were administered once daily on days 1, 8, 15, and 22 of cycle 1. If grade 2 or higher CRS occurred after the fourth epcoritamab administration during cycle 1, corticosteroids were given with epcoritamab for 4 days or until resolution of CRS occurred.

To ensure patient safety and to better characterize CRS, 24-hour inpatient monitoring was required for the first full epcoritamab dose.

The Protocol (online only) was approved by site-specific institutional review boards and/or institutional or central ethics committees before study initiation. The study was conducted in accordance with the International Council for Harmonisation E6 (R2) guidelines on good clinical practice and the principles of the Declaration of Helsinki. All patients reviewed and signed informed consent forms before enrollment.

### End Points and Assessments

The primary end point was overall response rate (ORR) by the independent review committee (IRC) using Lugano criteria.^9^ Secondary end points included duration of response (DOR), complete response (CR) rate, duration of CR, progression-free survival (PFS), time to response per IRC, and OS. Subgroup analyses were prespecified. In addition, minimal residual disease (MRD) was assessed by circulating tumor DNA using the clonoSEQ MRD assay (Adaptive Biotechnologies, Seattle, WA; Data Supplement). Safety end points included adverse events (AEs) and laboratory abnormalities. Relatedness of AEs to treatment was designated by the investigator.

Imaging assessments for efficacy (mandatory fluorodeoxyglucose positron emission tomography and either computed tomography or magnetic resonance imaging), along with MRD evaluation and physical examination, took place at weeks 6, 12, 18, 24, 36, and 48 and every 24 weeks thereafter.

AEs were coded using Medical Dictionary for Regulatory Activities (MedDRA) version 24.1, and severity was graded using National Cancer Institute Common Terminology Criteria for Adverse Events version 5.0. CRS and immune effector cell–associated neurotoxicity syndrome (ICANS) were graded using criteria from the American Society for Transplantation and Cellular Therapy criteria.^10^ Clinical tumor lysis syndrome was graded using criteria by Cairo-Bishop.^11^ Other assessments, including pharmacokinetics, antidrug antibodies, and patient-reported outcomes, are summarized in the Data Supplement.

### Statistical Analysis

Enrollment occurred in two stages; 28 patients with DLBCL were enrolled in the first stage. Interim analysis was conducted when approximately 25 patients with DLBCL had follow-up of up to 12 weeks. Futility stopping criteria were not met; therefore, an additional 100 patients with DLBCL were enrolled in the second stage, with up to 30 additional patients having other types of aggressive non-Hodgkin lymphomas.

ORR was defined as the proportion of patients who achieved best overall response of CR or partial response (PR). Best overall response per response criteria before initiation of subsequent antilymphoma therapy was summarized. The primary analysis of ORR was IRC-assessed response per Lugano criteria in the full analysis population (all patients who received at least one dose of epcoritamab). The ORR and the corresponding 95% exact CI were calculated.

PFS was defined as time from day 1 of cycle 1 to first documented disease progression or death because of any cause, whichever occurred earlier. Patients who remained alive without disease progression at the cutoff date were censored at the date of last evaluable disease assessment before the start of subsequent antilymphoma therapy. For patients who remained alive with incomplete or no baseline tumor assessment, PFS was censored on day 1 of cycle 1.

Time-to-event end points (DOR, PFS, and OS) were analyzed using Kaplan-Meier estimates (median time and 95% CI) with the number and percentage of patients with an event or censoring reported. Efficacy analyses were performed on the full analysis set; the safety analysis set was identical to the full analysis set (all patients who received at least one dose of epcoritamab). A landmark analysis was conducted for PFS by MRD status up to cycle 3 day 1 (day 60, considering ± 3-day window). Data were analyzed using SAS software version 9.4 (SAS Institute, Inc, Cary, NC; Data Supplement).

## RESULTS

### Patients and Treatment Exposure

Between June 19, 2020, and the data cutoff date of January 31, 2022, there were 157 patients who were enrolled at 54 global sites and treated with epcoritamab. At a median follow-up of 10.7 months, 106 patients discontinued study treatment: 83 (52.9%) because of disease progression, 11 (7.0%) because of AEs, seven (4.5%) because of decision to undergo allogeneic transplantation, four (2.5%) because of patient withdrawal, and one (0.6%) after achieving a PR to undergo CAR T-cell therapy. Demographic and baseline disease characteristics are given in Table 1. Patients had received a median of three prior lines of therapy (range, 2-11); 96 (61.1%) patients had primary refractory disease; 119 (75.8%) patients were refractory to two or more consecutive lines of therapy. The median time from initial diagnosis was 1.6 years (19 months). Sixty-one patients (38.9%) received prior CAR T-cell therapy, 46 (75.4%) of whom had progressive disease (PD) within 6 months of CAR T-cell therapy.

**TABLE 1.**
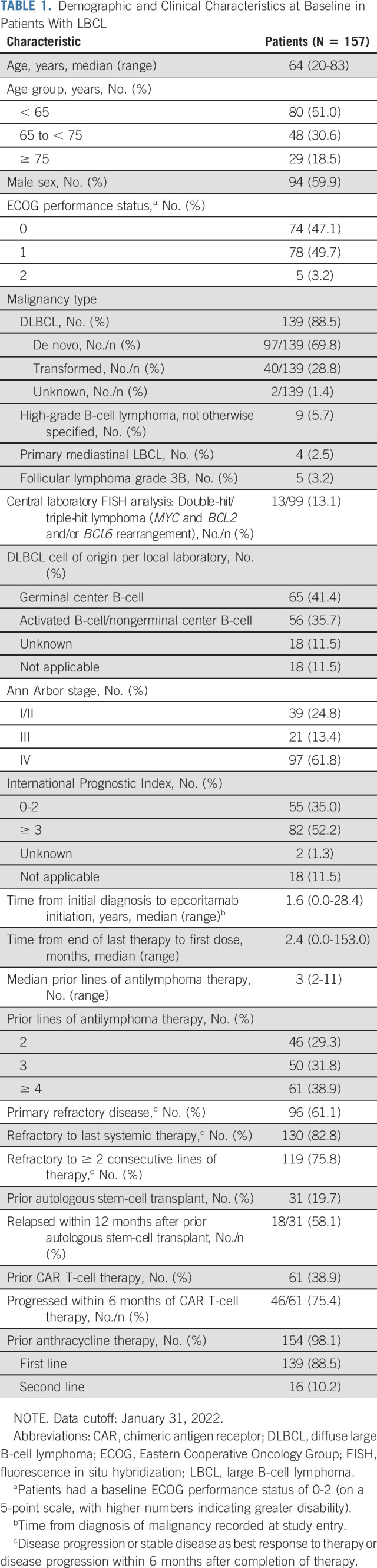
Demographic and Clinical Characteristics at Baseline in Patients With LBCL

Patients received a median of five cycles (15 doses) of epcoritamab therapy (range, 1-20). As of the data cutoff date, 51 patients (32.5%) continued receiving study treatment; 56.1% continued into the follow-up period.

### Efficacy

**FIG 1.**
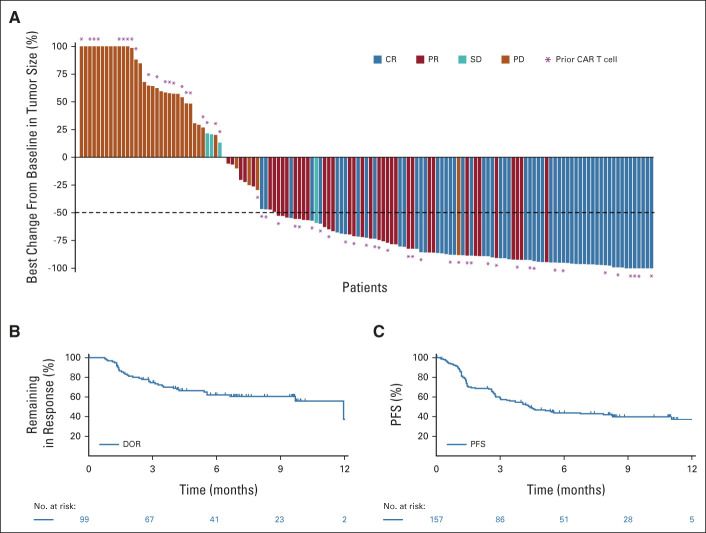
Efficacy results (per IRC; Lugano criteria) with epcoritamab in patients with LBCL. (A) shows best percentage change in sum-of-product perpendicular diameters of target lesions for patients with LBCL. Asterisks represent patients with prior exposure to CAR T-cell therapy. (B) shows the Kaplan-Meier curve for DOR. Results were similar in the DLBCL population (data not shown). (C) shows the Kaplan-Meier plot of PFS. A PFS ad hoc analysis using the Mantel-Byar approach showed a hazard ratio (95% CI) for patients with CR versus nonresponders of 0.11 (0.04 to 0.25) and a hazard ratio (95% CI) for patients with PR versus nonresponders of 0.47 (0.26 to 0.86). Thirty-six patients had disease progression (n = 28) or died (n = 8) within the first 6 weeks of treatment. Data cutoff: January 31, 2022. CAR, chimeric antigen receptor; CR, complete response; DLBCL, diffuse large B-cell lymphoma; DOR, duration of response; IRC, independent review committee; LBCL, large B-cell lymphoma; PD, progressive disease; PFS, progression-free survival; PR, partial response; SD, stable disease.

All 157 patients were efficacy and safety evaluable. The ORR per IRC using Lugano criteria was 63.1% (n/N = 99/157; 95% CI, 55.0 to 70.6), and the CR rate was 38.9% (n/N = 61/157; 95% CI, 31.2 to 46.9). Efficacy outcomes are summarized in Table 2. Best percentage changes from baseline in sum-of-product perpendicular diameters of target lesions are shown in Figure 1A. The median DOR (mDOR) per Kaplan-Meier estimates was 12.0 months in patients with LBCL (95% CI, 6.6 to not reached; Fig 1B). mDOR among complete responders was not reached. An estimated 88.7% of complete responders remained in response at 6 and 9 months. The median time to response was 1.4 months (range, 1.0-8.4). The median time to CR was 2.7 months (range, 1.2-11.1). Most CRs were achieved by the first or second assessment; however, nine patients converted from a PR to a CR at or after the week 36 tumor assessment (range, 32.3-48.1 weeks; Data Supplement).

**TABLE 2.**
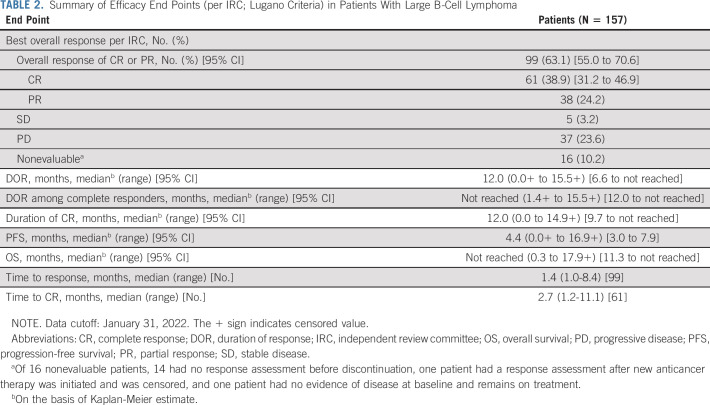
Summary of Efficacy End Points (per IRC; Lugano Criteria) in Patients With Large B-Cell Lymphoma

The median PFS was 4.4 months (95% CI, 3.0 to 7.9; Fig 1C), and the PFS rate at 6 months was 43.9% (95% CI, 35.7 to 51.7). Median PFS among complete responders was not reached (95% CI, 14.5 to not reached). Twenty-eight patients had disease progression within the first 6 weeks of treatment, and eight deaths occurred within the first 6 weeks of treatment; reasons for these early deaths included PD in five patients and AEs in three patients (one each with COVID-19 disease, hepatotoxicity in a patient with PD in the liver, and myocardial infarction). Median OS was not reached (95% CI, 11.3 to not reached; Data Supplement). Of 107 MRD-evaluable patients, 49 (45.8%) were MRD-negative (95% CI, 36.1 to 55.7). An estimated 78.7% of these patients remained MRD-negative at 6 months. Patients who achieved MRD negativity had longer PFS versus those who were MRD-positive (Data Supplement).

**FIG 2.**
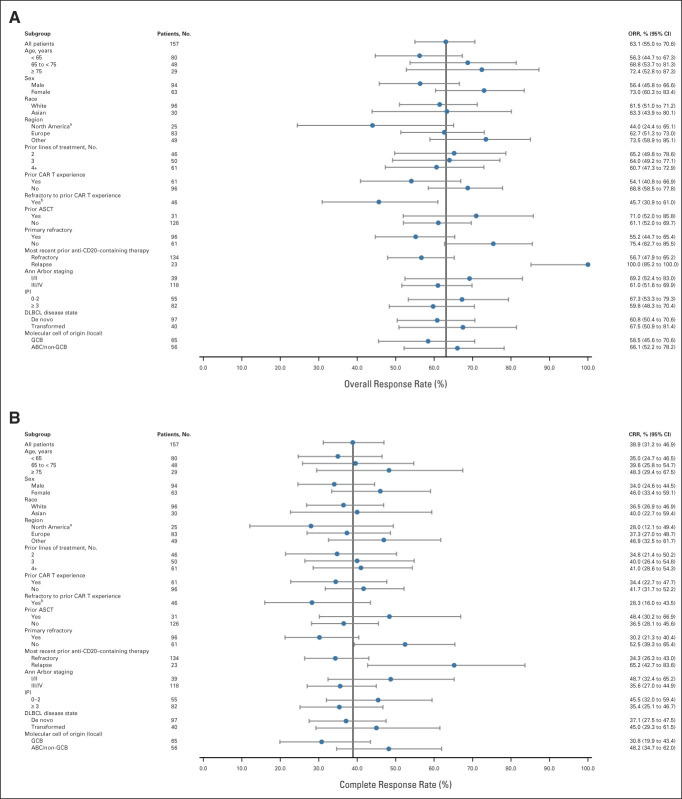
Response (per IRC; Lugano criteria) in prespecified subgroups of patients with LBCL. (A) ORRs. (B) CR rates. Data cutoff: January 31, 2022. ^a^A greater proportion of patients in North America had exposure to CAR T-cell therapy compared with other regions. ^b^Per the statistical analysis plan, subgroup analyses were not performed on subgroups with fewer than 20 patients. The ORR in patients who were not refractory to prior CAR T-cell therapy (n = 15) was 80.0% (95% CI, 51.9 to 95.7). The CR rate in patients who were not refractory to prior CAR T-cell therapy (n = 15) was 53.0% (95% CI, 26.6 to 78.7). ABC, activated B-cell; ASCT, autologous stem-cell transplantation; CAR, chimeric antigen receptor; CR, complete response; CRR, complete response rate; DLBCL, diffuse large B-cell lymphoma; GCB, germinal center B-cell; IPI, International Prognostic Index; IRC, independent review committee; LBCL, large B-cell lymphoma; ORR, overall response rate.

Concordance between the IRC and investigator assessments was high at 82.8% (kappa: 0.77 [95% CI, 0.69 to 0.84]; Data Supplement). For key subgroups, benefit with epcoritamab was consistent with that of the overall population (Figs 2A and 2B). In patients with primary refractory disease (n = 96), the ORR was 55.2% and the CR rate was 30.2%. In patients who received prior CAR T-cell therapy (n = 61), the ORR was 54.1% and the CR rate was 34.4%, with a mDOR of 9.7 months (95% CI, 5.4 to not reached); mDOR in patients with CR was not reached. In patients who did not receive prior CAR T-cell therapy (n = 96), the ORR was 68.8% and the CR rate was 41.7%, with a mDOR of 12.0 months (95% CI, 5.6 to not reached); mDOR in patients with CR was not reached. Of note, regional differences in ORRs and CR rates were observed, likely because of a higher proportion of patients with prior CAR T-cell exposure in North America.

### Safety

Treatment-emergent AEs observed with epcoritamab are summarized in Table 3. Grade 3 and higher AEs were observed in 61.1% of patients; treatment-related grade 3 and higher AEs were observed in 26.8% of patients. The most common treatment-related AEs were CRS (49.7%), injection site reaction (19.7%), and neutropenia (17.8%; Data Supplement). Most AEs (including treatment-related AEs) occurred in the first 12 weeks (cycles 1-3) of epcoritamab treatment, and the incidence of AEs declined after 12 weeks. Only one treatment-related serious AE occurred after week 12 (grade 1 CRS). Grade 3 or 4 infections are listed in the Data Supplement.

**TABLE 3.**
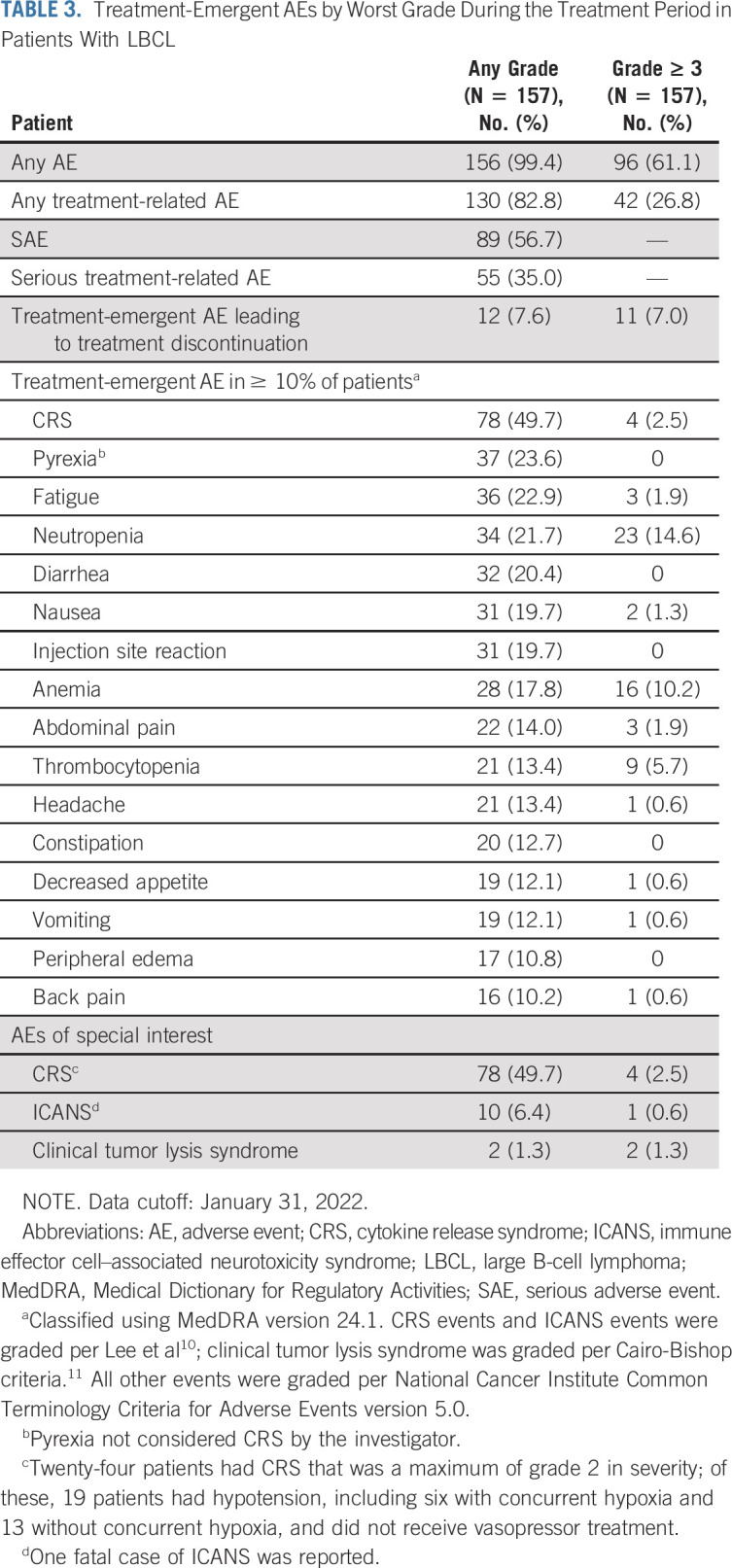
Treatment-Emergent AEs by Worst Grade During the Treatment Period in Patients With LBCL

Treatment-emergent AEs leading to discontinuation occurred in 12 patients (7.6%); three patients discontinued because of treatment-related AEs, including worsening of chronic lymphocytic inflammation with pontine perivascular enhancement responsive to steroids (see the description in the Data Supplement), CRS, and ICANS (one patient each). Nine patients (5.7%) had fatal treatment-emergent AEs, including COVID-19 disease in two patients and one case each with myocardial infarction, hepatotoxicity, progressive multifocal leukoencephalopathy, loss of consciousness, general health deterioration, pulmonary embolism, and ICANS. None of these AEs were considered related to epcoritamab by the investigator, except for the one fatal ICANS event, which had multiple concurrent confounding factors (Data Supplement). ICANS events occurred in 10 (6.4%) patients, including seven patients with grade 1, two patients with grade 2, and one fatal event. At least one event of CRS was observed in 49.7% of patients, mostly grade 1 in severity (n = 50; grade 2: n = 24; grade 3: n = 4); no grade 4 or 5 events were observed. Most CRS events occurred after the first full dose (Data Supplement) on day 15 of cycle 1 with a median time to onset of 0.8 days (20 hours). CRS resolved in 77 of 78 patients (98.7%); the median time to resolution from onset after first full dose was 2 days (48 hours). CRS was treated with tocilizumab in 22 (28.2%) patients and with corticosteroids (beyond those required for CRS prophylaxis) in 16 (20.5%) patients. Clinical tumor lysis syndrome occurred in two patients (grade 3 in severity) and was considered related to treatment. COVID-19–related events occurred in 10 (6.4%) patients; two of these cases were fatal but deemed to be unrelated to treatment.

The overall incidence of neutropenia was 21.7% (34 of 157). Febrile neutropenia was observed in four patients (2.5%) and was considered treatment-related in one patient (0.6%). Treatment with granulocyte colony-stimulating factor was required in 16 patients (10.2%).

### Patient-Reported Outcomes

Patients reported improvements in their lymphoma-related symptoms and overall quality of life during treatment. Clinically meaningful improvements were reported in the Functional Assessment of Cancer Therapy-Lymphoma scores (ie, lymphoma subscale, trial outcome index, Functional Assessment of Cancer Therapy-General total score, and Functional Assessment of Cancer Therapy-Lymphoma total score) and the EuroQol-5 Dimensions-3 Levels health utility score and EuroQol visual analog scale from day 1 of cycle 1 to day 1 of cycle 9 (Data Supplement).

## DISCUSSION

Subcutaneous epcoritamab achieved rapid, deep, and durable responses, including CRs and MRD negativity, in this cohort of patients with challenging-to-treat and highly refractory LBCL. Overall response and CR rates were 63.1% (95% CI, 55.0 to 70.6) and 38.9% (95% CI, 31.2 to 46.9), respectively. Responses were primarily observed early, by either the first or the second response assessment (scheduled at weeks 6 and 12). Among complete responders, mDOR was not reached at the time of analysis. Median PFS was not reached in complete responders, and median OS was not reached at the time of analysis. As of the data cutoff date, 32.5% of patients continued to receive study treatment and 56.1% continued into the follow-up period. This cohort represents a heavily pretreated, heterogeneous patient population. Patients had a median of three prior lines of therapy at a median of 19 months from diagnosis: 61.1% had primary refractory disease, 28.8% with DLBCL had transformed DLBCL, and 38.9% had received prior CAR T-cell therapy, 75.4% of whom were refractory to CAR T-cell therapy. The aggressive disease of this patient population was further demonstrated in that 36 patients had disease progression (n = 28) or died (n = 8) within the first 6 weeks. Responses to epcoritamab were consistent across several prespecified subgroups, including age, line of therapy, primary refractory disease, and prior exposure to CAR T-cell therapy. Forty-nine (45.8%) of 107 patients were MRD-negative per circulating tumor DNA analysis, with most (an estimated 78.7%) remaining MRD-negative after 6 months. Furthermore, MRD negativity was associated with longer PFS, highlighting the depth and durability of response to continuous epcoritamab treatment.

The safety profile of epcoritamab was consistent with previous reports. Notably, the majority of AEs occurred early (ie, in the first 12 weeks) in the treatment course. CRS, observed in 78 (49.7%) patients, was mostly low grade, predictable in terms of timing, and resolved. CRS occurred most frequently after the first full epcoritamab dose; tocilizumab was used to manage CRS in 22 of 78 (28.2%) patients. ICANS events were limited to mostly grade 1, and all resolved apart from the one fatal ICANS event in a patient with several confounding factors.

Results observed with epcoritamab in patients with relapsed or refractory LBCL favorably compare with those observed in approved antilymphoma immunotherapies, although differences in patient populations and study designs should be considered. CAR T-cell therapies (axicabtagene ciloleucel, tisagenlecleucel, and lisocabtagene maraleucel) demonstrated high ORRs (52%-82%) and CR rates (40%-54%) in phase I/II studies.^12-14^ However, many patients are ineligible for or do not receive CAR T-cell therapy because of rapidly progressing disease, complex manufacturing, limited accessibility, or patient preference.^4,15^ Notably, 38.9% of patients in the present study received prior CAR T-cell therapy, to which most were refractory; the number of patients in our study with prior CAR T-cell exposure is among the largest reported to date in LBCL. Among patients with relapsed or refractory DLBCL who received CAR T-cell therapy, a 43% risk of relapse has been shown and outcomes after relapse are poor.^16^ In the present study, epcoritamab showed clinical activity in patients who had received prior CAR T-cell therapy (n = 61), with an ORR of 54% and a CR rate of 34%. Numerically higher clinical activity was observed in patients without prior CAR T-cell therapy (n = 96), with an ORR of 69% and a CR rate of 42%. Recently approved therapies in the United States (polatuzumab vedotin plus bendamustine and rituximab, tafasitamab plus lenalidomide, selinexor, and loncastuximab tesirine) vary with regard to their mechanisms of action, safety profiles, and clinical activity.^17-22^ In the pivotal studies for polatuzumab vedotin plus bendamustine and rituximab and tafasitamab plus lenalidomide, which enrolled patients with fewer lines of prior therapy and few patients with primary refractory disease or CAR T-cell exposure, response rates were 60%-63% in patients ineligible for transplantation with at least one prior systemic regimen, and CR rates were 43%-50%.^17,19^ Despite shorter follow-up times, clinical activity observed with single-agent epcoritamab was comparable with that seen for CAR T-cell therapy and is favorable to other approved treatment options, considering the more refractory and difficult-to-treat population. Subcutaneous administration of epcoritamab may be a convenient alternative to intravenous therapies for both long-term and first-line use.

CD3xCD20 bispecific antibodies are a treatment modality being developed for treatment of non-Hodgkin lymphomas, with one compound approved in the European Union for relapsed or refractory follicular lymphoma.^23-26^ Responses with epcoritamab were shown to deepen from PR to CR at the week 36 assessment or later in nine patients, eight of whom had ongoing responses, thereby suggesting added clinical benefit with continuous treatment in a subset of patients.

Given the lack of direct comparison, there are currently no data to suggest whether the best outcome for heavily pretreated patients can be achieved by treating until disease progression or by stopping after a fixed number of treatment cycles. Treatment until progression ensures ongoing anti-CD20–directed, T-cell–mediated tumor suppression/surveillance. Continuation of subcutaneous epcoritamab at reduced frequency in later cycles may provide the greatest chance of durable remissions with limited burden for patients, but further studies would be helpful to determine the best treatment duration strategy for patients who achieve a CR. In this study, epcoritamab led to patient-reported improvements in their lymphoma-related symptoms and overall quality of life while receiving therapy. One limitation of our study is that it was a single-arm design with no control group for comparison.

Single-agent epcoritamab demonstrated a high ORR, including deep and durable CRs, in a challenging-to-treat and highly refractory patient population with relapsed or refractory LBCL. Efficacy was consistent across key subgroups. Epcoritamab was mostly well tolerated, with few discontinuations because of AEs. CRS was manageable, with predictable timing, and was mostly grade 1 or 2. As long-term treatment, epcoritamab is administered on a once monthly basis as a subcutaneous injection, making it an attractive and convenient off-the-shelf alternative to other antilymphoma immunotherapies. These results support ongoing and future clinical trials of epcoritamab both as monotherapy and in combination in late and earlier lines of treatment for B-cell non-Hodgkin lymphoma.

## Data Availability

Clinical trial data can be requested by qualified researchers for use in rigorous, independent scientific research as long as the trials are not part of an ongoing or planned regulatory submission. Sharing of data is subject to protection of patient privacy and respect for the patient's informed consent. The data will be provided following review and approval of a research proposal and Statistical Analysis Plan and execution of a Data Sharing Agreement. For approved requests, the data will be accessible for 12 months, with possible extensions considered. For more information on the process or to submit a request, contact clinicaltrials@genmab.com.
